# Efficacy of Restricting Dietary Protein Intake Combined with Buyang Huanwu Decoction in Treating Diabetic Nephropathy and Its Effect on Patients' Inflammatory Factor Levels

**DOI:** 10.1155/2021/5906244

**Published:** 2021-11-23

**Authors:** Dan Chen, Dan Huang, Taotao Hu, Fang Chen

**Affiliations:** Department of Nephrology, Wuhan No. 1 Hospital, Wuhan 430000, Hubei, China

## Abstract

**Objective:**

To study the efficacy of restricting dietary protein intake combined with Buyang Huanwu decoction in treating diabetic nephropathy (DN) and its effect on patients' inflammatory factor levels.

**Methods:**

The medical data of 150 DN patients treated in *Wuhan No.1 Hospital* (June 2018—May 2021) were retrospectively analyzed. All patients received regular therapy, those who received the intervention of restricting dietary protein intake were included in the control group (*n* = 75), and on this basis, those treated with Buyang Huanwu decoction were included in the experimental group (*n* = 75), so as to scientifically evaluate their efficacy and inflammatory factor levels after treatment.

**Results:**

The patients' general information was not statistically different between the two groups (*P* > 0.05); after treatment, the experimental group gained remarkably higher marked effective rate and total effective rate of treatment than the control group (*P* < 0.05); the inflammatory factor levels of all patients were obviously better than before (*P* < 0.05), and the levels of TNF-*α*, IL-2, IL-8, IL-4, and IL-10 were obviously lower in the experimental group than in the control group (*P* < 0.05); the levels of fasting blood glucose, 2 h postprandial blood glucose, and glycosylated hemoglobin of all patients were remarkably lower than before (*P* < 0.05), but with no significant between-group difference (*P* > 0.05); the renal function indexes of all patients were better than before, and between the two groups, the levels of 24 h microalbuminuria, 24 h urine protein excretion, and serum creatinine were obviously lower and the glomerular filtration rate was significantly higher in the experimental group (*P* all <0.05), and the patients' traditional Chinese medicine (TCM) symptom scores were remarkably lower in the experimental group (*P* < 0.05).

**Conclusion:**

Jointly applying Buyang Huanwu decoction on the basis of restricting dietary protein intake can effectively promote the clinical efficacy of DN, which is conducive to adjusting the inflammatory factor levels, promoting the patients' renal function, and alleviating the clinical symptoms.

## 1. Introduction

Diabetic nephropathy (DN), a microvascular complication of diabetes, is a chronic kidney disease caused by diabetes and an important trigger of renal failure [1, 2]. Its pathogenesis is not fully defined yet, but it is generally believed that blood glucose, blood pressure, and the course of the disease are all key factors leading to structural and functional damage to the kidney. DN is treated clinically by glycemic and blood pressure control and urinary protein reduction, including lifestyle interventions, correcting lipid metabolism disorders, and treating renal dysfunction [3–5]. Among them, lifestyle interventions aim to achieve glucose lowering and standard protein intake maintenance by controlling the patients' diet. The intervention of dietary protein intake restriction excludes the disadvantages of traditional patterns, reduces the patients' urinary protein excretion, and alleviates renal function impairment through restricting the dietary protein intake in their diet. In recent years, traditional Chinese medicine (TCM) therapy has shown great advantages in the clinical treatment of kidney diseases. Based on the TCM theory and combined with the pathogenesis features of DN, the author concluded that spleen-kidney deficiency and the syndrome of dampness-heat and blood stasis are the main pathogenic features of DN, while Buyang Huanwu decoction, which comes from *Yilin Gaicuo*, is a representative formula for invigorating qi and blood, which is mostly used to treat disease syndromes from qi deficiency and blood stasis, with efficacy that has been shown in clinical practice. Currently, there are few related studies on dietary protein restriction intervention and Buyang Huanwu decoction in curing DN, and the effectiveness of their combination remains controversial. Based on this, the study retrospectively analyzed 150 DN patients treated in our hospital to explore the efficacy of restricting dietary protein intake combined with Buyang Huanwu decoction for the treatment of DN.

## 2. Study Plan

### 2.1. Patient Screening and Grouping

The medical records of 150 DN patients treated in *Wuhan No.1 Hospital* (June 2018—May 2021) were retrospectively analyzed. All patients received the regular therapy, those who received the intervention of restricting dietary protein intake were included in the control group (*n* = 75), and on this basis, those treated with Buyang Huanwu decoction were included in the experimental group (*n* = 75). The study was approved by the ethics committee of *Wuhan No.1 Hospital*.

### 2.2. Inclusion Criteria

(1) The patients met the clinical diagnosis criteria for DN [6] as well as the dialectical diagnostic criteria for TCM syndromes [7]; (2) the patients were 30–75 years old; (3) the patients had normal cognitive function and communication function, and their medical records were complete; and (4) the patients and their family members signed the informed consent for the review of their relevant clinical data in this study.

### 2.3. Exclusion Criteria

(1) Patients treated with other therapies that affected observation during the same period; (2) patients with complicated severe urinary diseases, cardiovascular and cerebrovascular diseases or malignancies, etc.; (3) patients with type I diabetes mellitus; (4) patients who had to receive a low-protein diet, angiotensin-converting enzyme inhibitor (ACEI), or angiotensin II receptor antagonists; and (5) patients with unstable blood pressure.

### 2.4. Methods

After admission, all patients received routine treatment measures including diet control, exercise therapy, oral hypoglycemic agents, and subcutaneous injection of insulin for lowering the blood glucose, blood pressure reduction, lipid modification, and improvement of renal microcirculation [8].

Based on regular therapy, those in the control group accepted restricting dietary protein intake intervention. According to the food substitution method for diabetes mellitus, the dietician specified the balanced diet for the patients to rigorously follow, which contained 0.8 g/kg of protein with not less than half of high-quality protein, 104.6–125 kJ·kg^−1^·d^−1^ of calories, 25–30% of fat-supplied heat, and 50–60% of carbohydrate supply, cellulose, and inorganic salt, with the proportion of polyunsaturated fatty acid: monounsaturated fatty acid: saturated fatty acid = 1 : 1 : 1 [9, 10]. Additionally, those in the experimental group took the Buyang Huanwu decoction, and the formula was 60 g of Mongolian milkvetch root, 9 g of peony root, 9 g of Chinese angelica, 9 g of peach seed, 9 g of earthworm, 9 g of safflower, and 6 g of Sichuan lovage rhizome. All herbs were decocted with water, and the patients took one dose daily. The treatment cycle of all patients was 3 months.

### 2.5. Observation Indicators

Before treatment, the patients' general information including age, course of DN, course of diabetes, BMI, gender, complications, and human serum albumin (HSA) were recorded. The treatment effect of patients was evaluated by the *Guidelines for Clinical Research of New Drugs of Traditional Chinese Medicine (2014)* [11] and *Guidelines for Clinical Research of New Drugs of Traditional Chinese Medicine for Chronic Glomerulonephritis* [12]; i.e., compared to before treatment, 24 h urinary albumin excretion rate decreased by 50% and above was considered as marked effective, decreased by 20% and above but less than 50% as effective, and decreased by less than 20% or even increased as ineffective; and the total effective rate of treatment = (marked effective + effective)/total × 100%.

Patients' venous blood was extracted and placed under room temperature for 30 min and then centrifuged for 10 min under 3,000 r/min to take the supernatant for standby application, and the patients' tumor necrosis factor-*α* (TNF-*α*), interleukin-2 (IL-2), IL-4, IL-8, and IL-10 were measured by enzyme linked immunosorbent assay (ELISA). Patients' fasting blood glucose and 2 h postprandial blood glucose before and after treatment were measured by the glucose oxidase method with the automatic biochemical analyzer; the glycosylated hemoglobin level was measured by cation exchange-high-performance liquid chromatography (HPLC) with the VARIACT Type II Glycosylated Hemoglobin Analyzer. The patients' 24 h microalbuminuria and 24 h urine protein excretion levels were measured by radiometric analysis, the serum creatinine was measured by the sarcosine oxidase method, and the glomerular filtration rate was detected.

The scores of TCM clinical symptoms were evaluated with the *Guidelines for Clinical Research of New Drugs of Traditional Chinese Medicine* [13], mainly including fatigue, short breath and no desire to speak, lumbago, chest pain, back pain, pain worsening at night, limb numbness, dark-colored tongue, wiry pulse, ecchymosis, and astringent pulse. On a scale of 0–3 points, each clinical symptom was classified as none, occasional, often, and continuous.

### 2.6. Statistical Processing

The differences in study data were calculated with SPSS22.0, the pictures were drawn with GraphPad Prism 7 (GraphPad Software, San Diego, USA), the items included were enumeration data and measurement data, which were expressed by [*n* (%)] and ($\bar{x}$ ± *s*) and examined by the *X*^2^ test and *t* test, respectively, and differences were considered statistically significant at *P* < 0.05.

## 3. Results

### 3.1. General Information

The patients' general information was not statistically different between the two groups (*P* > 0.05), which met the study criterion of controlled experiment, see Table 1.

### 3.2. Clinical Efficacy

**Figure 1:**
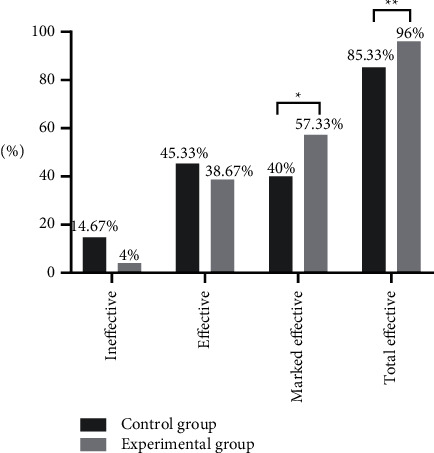
Between-group comparison of clinical efficacy (%). Note: the horizontal axis indicated the evaluation dimensions, and the vertical axis indicated the percentage (%); the numbers of ineffective cases, effective cases, and marked effective cases in the control group were 11, 34, and 30, respectively, and the number of total effective cases was 64; the numbers of ineffective cases, effective cases, and marked effective cases in the experimental group were 3, 29, and 43, respectively, and the number of total effective cases was 72; ^*∗*^indicates a significant between-group difference in the marked effective rate of treatment (*X*^2^ = 4.510, *P*=0.034), and ^*∗∗*^indicates a significant between-group difference in the total effective rate of treatment (*X*^2^ = 5.042, *P*=0.025).

Between the two groups, the marked effective rate and total effective rate of treatment were greatly higher in the experimental group (*P* < 0.05), presenting statistical significance, see Figure 1.

### 3.3. Levels of Inflammatory Factors

After treatment, the levels of inflammatory factors in patients of both groups were obviously better than before (*P* < 0.05), and the levels of TNF-*α*, IL-2, IL-8, IL-4, and IL-10 were obviously lower in the experimental group than in the control group (*P* < 0.05), see Table 2.

### 3.4. Blood Glucose Levels

After treatment, the levels of fasting blood glucose, 2 h postprandial blood glucose, and glycosylated hemoglobin in patients of both groups were significantly lower than before (*P* < 0.05), but with no significant between-group differences (*P* > 0.05), see Table 3.

### 3.5. Renal Function

After treatment, various renal function indicators of patients in both groups were better than before, and the patients in the experimental group obtained obviously lower levels of 24 h microalbuminuria, 24 h urine protein excretion, and serum creatinine and significantly higher glomerular filtration rate (*P* all <0.05) than the control group, see Table 4.

### 3.6. Scores of TCM Clinical Symptoms

**Figure 2:**
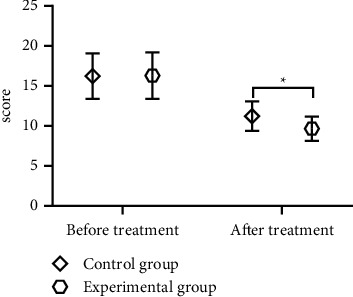
Between-group comparison of patients' scores of TCM clinical symptoms ($\bar{x}$ ± *s*). Note: the horizontal axis indicates before and after treatment, and the vertical axis indicates the scores; before and after treatment, the scores of TCM clinical symptoms of the control group were (16.24 ± 2.85) and (11.24 ± 1.83), and those of the experimental group were (16.30 ± 2.92) and (9.67 ± 1.51), respectively; and ^*∗*^ indicates the significant between-group difference in the scores of TCM clinical symptoms after treatment (*t* = 5.701, *P* < 0.001).

Compared with the control group after treatment, the scores of TCM clinical symptoms in patients of the experimental group were obviously lower (*P* < 0.05), with statistical significance, see Figure 2.

## 4. Discussion

Recently, relevant studies have shown that the occurrence of type II diabetes and its common complications is closely related to the inflammatory response, and tumor necrosis factor-*α* and interleukin factors are clinically common inflammatory response markers that can effectively predict the incidence of type II diabetes [14–16]. The study by Ma et al. [17] showed that the decreased glomerular filtration rate and increased serum creatinine level were usually accompanied by an obvious increase in the levels of inflammatory factors, and thus, the acute inflammatory response in type II diabetic patients was strongly linked to the emergence and progression of DN. Besides, the chronic inflammatory response intensity also parallels the degree of renal damage in diabetes; with higher urinary protein levels, the inflammatory response in patients is more severe. It follows that the increased levels of inflammatory factors in serum of DN patients are the important factor for the development of diabetic microangiopathy, and thus, anti-inflammatory therapy will also be a novel strategy for preventing, monitoring, and treating type II diabetes and its complications [18, 19]. Based on relevant TCM studies, it seems that the occurrence of DN may be due to the deficiencies of qi and yin caused by the disease with the symptom of frequent drinking and urination, in which the deficiency of yin is the primary aspect and the dryness-heat is the secondary aspect. Over time, the deficiency of yin involves qi and causes qi-yin deficiency, and the dryness-heat will be recessed gradually. Qi deficiency causes week blood circulation, while yin deficiency causes obscure bloodstream, and either one of the results in unsmooth blood flow, stasis, and blocking the meridians, forming the syndrome of blood stasis due to qi deficiency. The occurrence and development of DN show a dynamic evolution process, and finally, spleen-kidney deficiency is mingled with damp-heat and blood stasis [20–22]. Therefore, invigorating the spleen and kidney and promoting blood circulation to remove blood stasis are the root for treatment, and Buyang Huanwu decoction, a classic TCM formula for qi invigoration and blood activation, is exactly a perfect choice for symptomatic treatment of syndromes caused by qi deficiency and blood stasis. A great amount of Mongolian milkvetch root in the formula can invigorate the primordial qi and then promote the circulation of blood. The study by Yin et al. [23] showed that Mongolian milkvetch root could reduce the urine protein excretion of DN patients, which might be related to the fact that the herb is associated with the regulating effect of renal cortex IV collagen and transforming growth factor-*β* in diabetes patients. In addition, peony root, Sichuan lovage rhizome, Chinese angelica, peach seed, and safflower are effective in promoting blood circulation to remove blood stasis, and combined with the results of modern pharmacological experiment, the first three also have the effect of dilating blood vessels and improving microcirculation. Also, earthworm in the formula has the moving and fleeing nature, so it can exert its effect of promoting blood circulation for removing obstruction in collaterals. Hence, the decoction mainly reduces proteinuria, improves the renal function, and then, treats DN by invigorating qi, promoting blood circulation and dredging channels.

In the author's opinion, medical nutrition intervention is also an essential part in the treatment of DN patients, the key point of which is to promote glomerular vasodilation through suitable protein intake and to increase the synthesis of prostaglandin E2 and prostaglandin F1_*α*_. In addition, the study by Couteur et al. [24] reported that suitable protein intake could increase glucagon secretion, which could control patients' blood glucose to a certain extent and improve clinical symptoms. Therefore, in this study, all enrolled patients accepted the intervention of restricting dietary protein intake and those in the experimental group received Buyang Huanwu decoction therapy additionally, and by comparing various clinical indicators, the results showed that the experimental group gained greatly higher marked effective rate and total effective rate of treatment than the control group (*P* < 0.05); after treatment, the levels of inflammatory factors in all patients were better than before (*P* < 0.05), and the levels of TNF-*α*, IL-2, IL-8, IL-4, and IL-10 were obviously lower in the experimental group than in the control group (*P* < 0.05); after treatment, the levels of fasting blood glucose, 2 h postprandial blood glucose, and glycosylated hemoglobin were significantly lower than before (*P* < 0.05), but with no significant between-group differences (*P* > 0.05), which was consistent with the report by Liu et al. [25]; various renal function indicators of all patients were better than before, and the experimental group obtained obviously lower levels of 24 h microalbuminuria, 24 h urine protein excretion, and serum creatinine and significantly higher glomerular filtration rate (*P* all <0.05) compared with the control group after treatment, the scores of TCM clinical symptoms were significantly lower in the experimental group (*P* < 0.05). It can be concluded from these results that implementing the restriction on dietary protein intake showed high efficacy in controlling inflammatory responses, reducing urine protein amount, and improving kidney function, and the combined application of Buyang Huanwu decoction could further improve the efficacy in DN patients, especially on the regulation of inflammatory factor levels. Besides, either treatment did not generate adverse reaction on patients' blood glucose control.

In conclusion, the combined application of Buyang Huanwu decoction and restricting dietary protein intake can effectively improve the clinical efficacy of DN, which is beneficial for adjusting the levels of inflammatory factors, improving the renal function of patients, and alleviating clinical symptoms. However, restricting dietary protein intake in this study was limited by the implementation cost and practice, and the clinical application program still needs further exploration on nutrition recipe, patient cooperation, supervision, and habit formation to ensure the effect of the program.

## Figures and Tables

**Table 1:** Between-group comparison of patients' general information (*n* = 75).

Indicator	Control	Experimental	*X* ^2^/*t*	*P*
Age (years)	61.58 ± 7.24	61.93 ± 7.38	0.293	0.770
Course of DN (years)	1.36 ± 0.44	1.35 ± 0.51	0.129	0.898
Course of diabetes (years)	8.84 ± 3.15	8.96 ± 3.22	0.231	0.818
BMI (kg/m^2^)	22.28 ± 1.23	22.56 ± 1.27	1.372	0.172
Gender			0.118	0.731
Male	50 (66.67)	48 (64)		
Female	25 (33.33)	27 (36)		
*Complication*				
Hypertension	36 (48)	33 (44)	0.242	0.623
Hyperlipidemia	25 (33.33)	27 (36)	0.118	0.731
Educational level			0.242	0.623
Junior high school and below	39 (52)	42 (56)		
Above junior school	36 (48)	33 (44)		
Serum albumin (g/L)	52.85 ± 6.22	53.57 ± 7.36	0.647	0.519

**Table 2:** Between-group comparison of levels of inflammatory factors (ng/L, $\bar{x}$ ± *s*).

Indicator		Control (*n* = 75)	Experimental (*n* = 75)	*t*/*P*
TNF-*α*	Before	31.75 ± 6.83	32.06 ± 6.79	0.279/0.781
	After	24.34 ± 5.12^*∗*^	15.23 ± 3.11^*∗*^	13.170/<0.001
IL-2	Before	24.87 ± 4.76	25.31 ± 5.42	0.528/0.598
	After	19.02 ± 3.46^*∗*^	12.14 ± 2.05^*∗*^	14.815/<0.001
IL-4	Before	44.27 ± 5.86	43.85 ± 5.24	0.463/0.644
	After	31.58 ± 5.02^*∗*^	21.23 ± 3.41^*∗*^	14.770/<0.001
IL-8	Before	46.92 ± 8.35	47.27 ± 8.26	0.258/0.797
	After	33.85 ± 6.11^*∗*^	18.45 ± 4.27^*∗*^	17.892/<0.001
IL-10	Before	50.26 ± 5.24	50.34 ± 3.01	0.115/0.909
	After	35.67 ± 6.51^*∗*^	25.83 ± 6.39^*∗*^	9.342/<0.001

Note: ^*∗*^significant differences in the levels of inflammatory factors before and after treatment in the same group (*P* < 0.05).

**Table 3:** Between-group comparison of patients' blood glucose levels ($\bar{x}$ ± *s*).

Indicator		Control group	Experimental group	*t*/*P*
Fasting blood glucose (mmol/L)	Before treatment	8.74 ± 1.46	8.85 ± 1.52	0.452/0.652
	After treatment	6.46 ± 0.79^*∗*^	6.33 ± 0.75^*∗*^	1.034/0.303
2 h postprandial blood glucose (mmol/L)	Before treatment	11.65 ± 2.07	12.04 ± 2.12	1.140/0.256
	After treatment	7.15 ± 1.02^*∗*^	7.22 ± 1.01^*∗*^	0.422/0.673
Glycosylated hemoglobin (%)	Before treatment	7.20 ± 0.88	7.17 ± 0.86	0.211/0.833
	After treatment	5.17 ± 0.58^*∗*^	5.25 ± 0.62^*∗*^	0.816/0.416

Note: ^*∗*^significant differences in the levels of blood glucose indicators before and after treatment in the same group (*P* < 0.05).

**Table 4:** Between-group comparison of patients' renal function indicators ($\bar{x}$ ± *s*).

Observation indicator		Control group	Experimental group	t/P
24 h microalbuminuria (mg/24 h)	Before treatment	237.45 ± 26.14	238.85 ± 26.44	0.326/0.745
	After treatment	116.08 ± 14.63^*∗*^	101.67 ± 15.24^*∗*^	5.907/<0.001
24 h urine protein excretion (g/24 h)	Before treatment	0.38 ± 0.08	0.39 ± 0.09	0.719/0.473
	After treatment	0.24 ± 0.05^*∗*^	0.15 ± 0.04^*∗*^	12.173/<0.001
Serum creatinine (*μ*mol/L)	Before treatment	93.47 ± 8.26	93.24 ± 8.30	0.170/0.865
	After treatment	75.86 ± 6.64^*∗*^	62.33 ± 6.15^*∗*^	12.947/<0.001
Glomerular filtration rate (ml/min)	Before treatment	86.54 ± 7.34	86.81 ± 7.50	0.223/0.824
	After treatment	103.16 ± 8.48^*∗*^	113.75 ± 9.26^*∗*^	7.304/<0.001

Note: ^*∗*^significant differences in the indicators before and after treatment of the same group (*P* < 0.05).

## Data Availability

Data to support the findings of this study are available on reasonable request from the corresponding author.
